# Digital Twin-Based Virtual Simulation Method for Visualization Monitoring of Flexible Production Lines Towards Intelligent Manufacturing

**DOI:** 10.3390/s26216929

**Published:** 2026-11-02

**Authors:** Yang Yi, Anqi Zhang, Huanlian Liu, Yi Lu, Di Jiang, Bin Wu

**Affiliations:** 1 College of Mechanical and Electronic Engineering, Nanjing Forestry University, Nanjing 210037, China; 2 School of Mechanical Engineering, Southeast University, Nanjing 211198, China; 3 College of Electronic and Optical Engineering, Nanjing University of Posts and Telecommunications, Nanjing 210096, China; 4 Nanjing Research Institute of Electronic Engineering, Nanjing 210008, China

**Keywords:** flexible production lines, digital twin, virtual simulation, 3D visualization monitoring, virtual-real synchronization

## Abstract

With the in-depth development of intelligent manufacturing worldwide, flexible production modes and corresponding production lines for personalized, customized, small-batch, and multi-variety products have become the dominant force in today’s manufacturing industry. To address the challenges of real-time information interaction and visualization monitoring in the flexible production lines (FPLs), this paper conducts in-depth research on the 3D visualization simulation of FPLs based on digital twin (DT) according to the characteristics of FPLs and the demand for intelligent development. Firstly, a five-layer DT framework for 3D visualization monitoring of FPLs is proposed, which is divided into the physical entity layer, twin model layer, twin data layer, application service layer, and connection transport layer. The corresponding DT models of the twin model layer are then organized at the atomic, unit, and system levels and described from four dimensions: geometric physical, attribute information, behavioral logic, and constraint rules. On this basis, the data interconnection mechanism, real-time data-driven twin model mapping, and twin data management methods of the above DT framework are further studied. Finally, a representative machining FPL for mechanical processing is selected as a case study, a virtual simulation system of FPLs for 3D visualization monitoring based on DT is developed and implemented. Through the implementation and application in the field operation, the effectiveness and feasibility of the proposed method and developed system are verified.

## 1. Introduction

With the rapid development of the new-generation information and communication technologies (New-ICT) and their successive application in the manufacturing industry, the entire industrial production system has been elevated to a higher level, driving the industrial revolution to enter a new stage [1,2,3]. How to integrate the New-ICT (such as the Internet of Things, big data, artificial intelligence) with manufacturing to meet the industry’s development needs in terms of socialization, personalization, service orientation, and greenness, has become a research hotspot in the development of intelligent manufacturing [4,5,6]. In the face of the current development opportunities in the manufacturing industry, the transformation and upgrading of the manufacturing industry have received great attention from all countries. These corresponding national manufacturing strategies have been proposed, such as the Industrial Internet in the United States, Industry 4.0 in Germany, and the Made in China 2025 strategy. Moreover, during the development of the manufacturing industry, manufacturing modes (such as cyber-physical integration manufacturing, “Internet+” manufacturing, cloud manufacturing and so on) have been proposed to assist in achieving the strategic goal of intelligent manufacturing [7,8].

To meet the production demands of small batches and multiple varieties of products, flexible production lines (FPLs) have emerged [9,10,11]. Currently, FPLs have largely achieved automation and flexibility on the shop-floor. However, there are still problems such as insufficient and incomplete information interaction and weak visualization monitoring capability, which lead to the insufficient monitoring of the execution information by workshop managers, seriously restricting the intelligent development of FPLs. The rapid development of New-ICT has provided a large amount of real-time data at the workshop level for various application systems of FPLs. How to manage and apply the real-time data, and further realize intuitive, transparent and real-time visualization monitoring of FPLs is an engineering problem that these intelligent manufacturing enterprises urgently need to solve [12]. For a long time, under the existing manufacturing mode, virtual simulation technology has been a key research focus in the visualization monitoring development of the FPL. These traditional virtual simulation methods mainly focus on two aspects [13,14]: one is to conduct virtual simulation for a certain dimension of one device, such as performing geometric-level motion simulation for a computer numerical control (CNC) machine or a robotic arm through trajectory planning; the other is to conduct digital production layout and virtual simulation for the shop-floor, and to provide virtual display and verification of the layout scheme of FPLs. However, the above traditional virtual simulation methods rarely interconnect with the physical space. During virtual simulation, these methods mainly rely on given design data and models, but do not adequately consider the behaviors, rules, and constraints associated with equipment, workshop, factories, etc., as well as the real-time dynamic data generated in the physical space. These methods lack a multi-dimensional integrated model that combines geometry, behavior, rule, and constraint, resulting in the current virtual simulation of the manufacturing process having the problems of “being virtual but not real, and being simulated but not true” [15].

Due to the fact that traditional virtual simulation technology is mainly applied to the pre-production layout planning of workshops and the optimization of production lines, it has limitations in the real-time visualization monitoring of FPLs. However, the emergence of digital twin (DT) technology can precisely bridge the gap of interconnection between virtual and physical spaces [16,17,18]. DT technology refers to the process and method of using digital technology to describe and model the characteristics, behaviors, formation processes and performance of physical world entities. The corresponding integrated DT model can be constructed, which integrates multi-disciplinary, multi-physical, multi-scale and dynamic probabilities, realizing real-time mapping and highly mirroring between the virtual and physical spaces. It can be used to simulate, monitor, diagnose, predict and control the formation process, state and behavior of physical entities in the real environment [19]. Therefore, through the deep integration of virtual simulation technology and DT technology, it is possible to achieve very realistic model construction in the virtual simulation process and the mapping behavior of real-time interaction between the virtual and real environments, thereby providing technical support for the real-time visualization monitoring of FPLs.

Currently, experts and scholars at home and abroad have conducted a large amount of research and practice on issues such as workshop data collection and monitoring models, visualization monitoring, digital twin shop-floor (DTS) and so on [20,21]. For example, Mori and Fujishima [22] developed a status monitoring and maintenance system for remote monitoring of machine tools, and commercial applications have been achieved. Zhang et al. [23] integrated real-time data during the process of modeling the workshop using hierarchical-timed-colored Petri net with decision tree algorithm, and dynamically presented the real-time status of the workshop. Li et al. [24] developed a novel RFID-based tracking and monitoring approach of real-time data to solve these production emergencies (e.g., new manufacturing tasks, facilities failure and tasks change) in production workshop. Furthermore, in terms of visualization monitoring of the workshop, the 3D visualization monitoring based on virtual simulation can provide more information about the workshop compared with the 2D monitoring, thereby enhancing the authenticity and transparency of workshop monitoring. Currently, the 3D visualization monitoring is widely applied in CNC machine tools and robotics, while its application in digital workshops, FPLs and DTS is still in the process of development. For example, aiming at the application requirements of virtual simulation monitoring of typical CNC machine tools, Hu et al. [25] presented a development architecture for a virtual simulation monitoring and processing optimization system that integrates data, models, communication, and optimization, to support the realization of intelligent manufacturing services. Jiang et al. [26] constructed an interactive, controllable and computable virtual workshop from three dimensions (i.e., visualization, logic and data), and elaborated on the evolution and operation mechanism of DTS, providing a reference for the promotion and applications of DTS in manufacturing fields. Zhang et al. [27] proposed a data-driven smart management and control framework of a DTS to solve the problems of strong uncertainty, dynamics, and high complexity during the operation process in a discrete manufacturing workshop. Yu et al. [28] proposed a method of real-time monitoring and dynamic bidirectional interaction in workshops based on the digital twin, so as to realize high-fidelity mapping from the virtual workshop to the physical workshop.

The above studies demonstrate important progress in data acquisition, virtual modeling, visualization, and monitoring management of the FPL. However, from the perspective of 3D visualization monitoring of an entire FPL, there remains a need to explicitly organize the physical production resources, hierarchical models, multi-source heterogeneous data, application services, and communication interfaces within a unified DT implementation framework. In particular, the mapping among these different levels of an entire FPL and the simultaneous representation of different granularity information require further systematic integration for continuous bidirectional virtual-real synchronization and visualization monitoring. Accordingly, in order to address these aforementioned problems, this paper proposes a DT framework for real-time 3D visualization monitoring of FPLs, and presents the corresponding construction strategies and methods of DT model for FPLs. Rather than introducing an entirely new set of functional components, the main contribution of this work lies in the scenario-oriented integration and operationalization for real-time 3D visualization monitoring for FPLs. Based on this, a digital twin-based virtual simulation system platform for FPLs is developed and implemented on a typical FPL of mechanical processing to demonstrate the feasibility of the proposed DT framework. Through the collection and transmission of real-time data in the physical space, the synchronous operation with the physical entity layer can be achieved, providing functions such as 3D visualization monitoring of real-time production status, data statistical analysis and management for FPLs. Therefore, this paper establishes a concrete implementation path from physical production data to hierarchical twin modeling and virtual monitoring, providing a practical basis for applying DT technology to real-time 3D visualization monitoring of FPLs.

The rest of this paper is organized as follows: Section 2 proposes the establishment of a DT framework for 3D visualization monitoring of FPLs; Section 3 presents the DT model construction method of the twin layer in the proposed DT framework; Section 4 summarizes the key technologies of virtual monitoring driven by real-time data for FPL; Section 5 elaborates the case study and demonstrates the application effects of the DT-based virtual simulation system in a typical FPL for mechanical processing. Finally, conclusions and future work are presented in Section 6.

## 2. A Digital Twin Framework for Visualization Monitoring of Flexible Production Lines

The FPL emerged based on the traditional combination machine tools in response to the demand for automation in the manufacturing industry. It has carried out numerical control (NC) on various process-functional combination machine tools and adopted computer control and management. The FPL not only retains the modular structure and high-efficiency processing characteristics of the combination machine tools, but also incorporates the flexible features of NC technology. The FPL mainly consists of the production processing system, the logistics and storage system, and the supporting information control system. Due to the clear hierarchical division of the FPL, it can be classified into atomic level, unit level and system level based on the composition of the production line in physical space. Among them, the atomic level is the lowest layer of the FPL, consisting of various equipment (such as machining equipment, storage equipment, logistics equipment, etc.) and auxiliary tools (such as pallets, rails, cutting tools, etc.); the unit level is composed of multiple atomic-level equipment, auxiliary tools and materials, thus forming a production unit that can execute a certain processing technology or has a certain processing capability; the system level is the entire production system composed of the above production unit levels, and can allocate tasks to the production unit levels according to the production order and achieve the production processing tasks of the FPL in an efficient and stable manner. These characteristics of the FPL provide a good foundation for constructing the DT framework of FPL.

**Figure 1:**
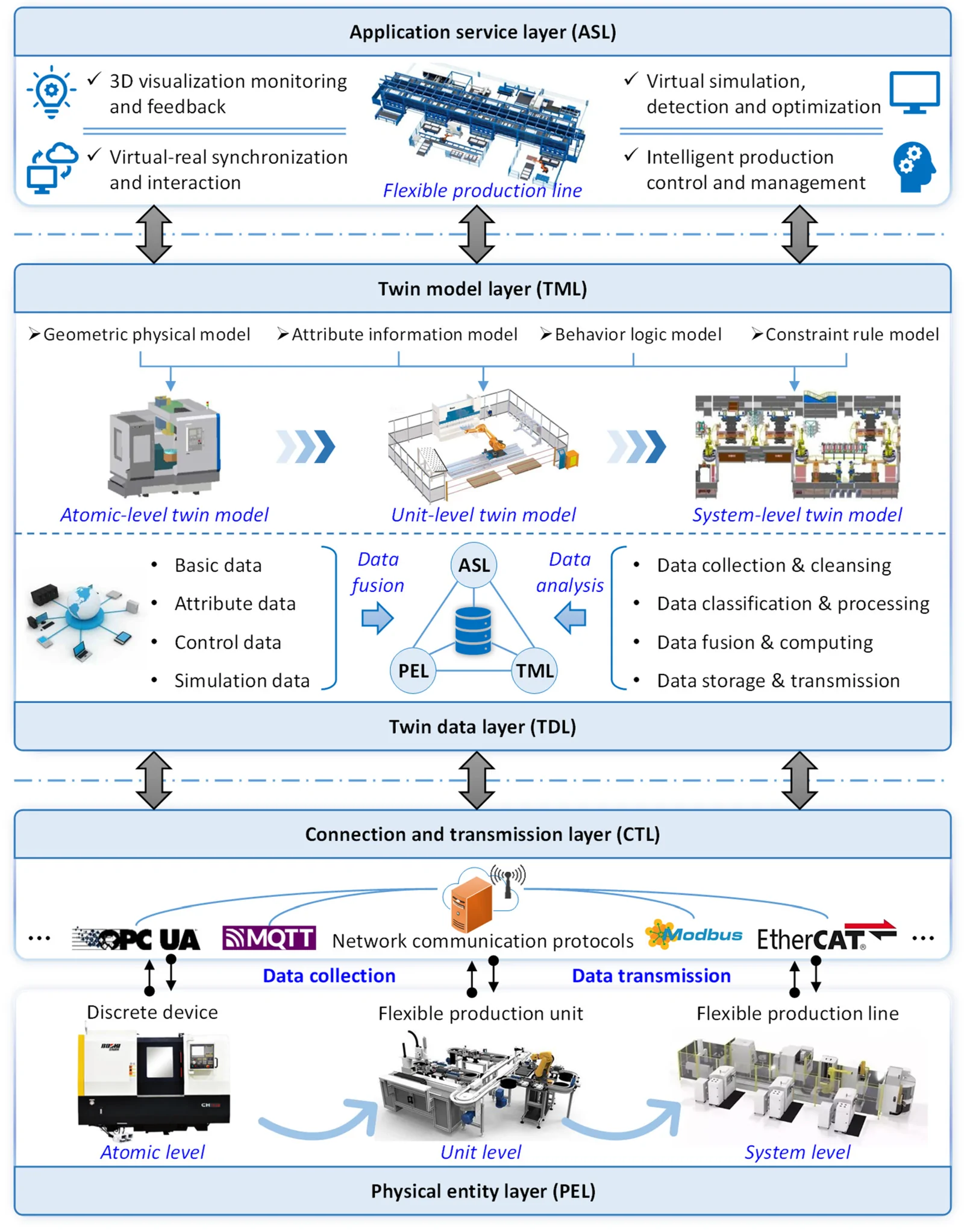
Structure diagram of a DT framework for 3D visualization monitoring of FPLs.

This paper is inspired by the DTS and the five-dimensional digital twin model proposed by Professor Tao Fei’s team at Beihang University [29,30,31]. Based on the development demand for real-time visualization monitoring of FPLs, a DT framework for 3D visualization monitoring of FPLs is proposed in this paper, as shown in Figure 1. It is divided into five layers in total, namely the physical entity layer (PEL), the twin model layer (TML), the twin data layer (TDL), the application service layer (ASL), and the connection and transmission layer (CTL).

### 2.1. Physical Entity Layer

The PEL refers to the collection of all physical entities involved at the atomic, unit, and system levels within an FPL. The atomic level consists of discrete devices, tools, and material entities within the production line. The unit level is composed of multiple atomic-level entities that can independently complete a certain processing technology or production task, such as CNC turning processing unit, a CNC milling processing unit, a cleaning unit, a measurement unit, etc. The system level is composed of all production units within the production line and the related computer information system and control system, responsible for coordinating and cooperating within the production line, decomposing tasks, and scheduling production, and ultimately completing production orders. The PEL is the foundation of the DT system framework for FPLs, and the research of other layers must be carried out based on the PEL.

### 2.2. Twin Model Layer

The TML is based on the PEL and constructs digital models that correspond to the physical entities within the scope of the monitoring tasks in the PEL. It not only maintains consistency in geometry and appearance, but also contains all the information, knowledge, and data of the PEL. This paper organizes the twin models at the atomic, unit, and system levels and describes each level using four dimensions: geometric physical model, attribute information model, behavior logic model and constraint rule model. Among them, the geometric physical model describes the size, shape, position, and modeled assembly or motion relationships of a physical entity; the attribute information model describes the basic attributes of the physical entity based on the basic characteristics and connection methods of the PEL; the behavior logic model describes the kinematic behavior and operational logic of the physical entity; the constraint rule model describes the constraint rules for the operation of the PEL, such as production process rules, production decision rules, etc., so that these constructed rules can be used to evaluate and optimize the FPL.

### 2.3. Twin Data Layer

The TDL is composed of PEL data, TML data, ASL data, and the fused data based on the above data. Among them, the PEL data include the basic data collected by sensors and the real-time data from the production control system; the TML data mainly consists of the basic data of the twin model and the simulation data generated during the virtual simulation process; the ASL data are generated by various application services through further calculation and analysis of the data; the fused data are the new data generated through data fusion based on the above data.

### 2.4. Application Service Layer

The ASL is supported by the TDL and provides corresponding application services for machining, logistics, maintenance, etc., in the production process of the FPL. It mainly drives the relevant application service functions of the FPL based on twin data and twin models, such as virtual-real synchronous interaction, simulation optimization, data statistics and analysis, etc., thereby providing technical support for the real-time visualization monitoring of the FPL.

### 2.5. Connection and Transmission Layer

The CTL is the key layer for achieving interconnection and communication among all layers of the DT framework. Among them, the connection between the PEL and other layers is the most important part in the entire CTL, which can be realized through communication protocols (such as Modbus, OPC UA, MQTT, etc.) or software development packages to achieve data collection and feedback from the PEL; the connection between the TDL and other layers is achieved through database access interfaces for data storage and output; the connection among various applications in the ASL is realized by defining unified data interfaces during the development of each application, thereby enabling data sharing.

## 3. Digital Twin Model Construction Method of the Twin Model Layer

The TML mainly consists of different DT models of the FPL, which is the foundation for achieving real-time visualization monitoring of the FPL. How to efficiently and reasonably construct the twin model (TM) plays a decisive role in the DT degree of the FPL. This paper constructs the TM of the FPL from four dimensions, i.e., geometry physics, attribute information, behavior logic, and constraint rule. Its formal expression is as follows:

*TM* = {*GPM*, *AIM*, *BLM*, *CRM*} (1)

where *GPM* is the geometric physical model, *AIM* is the attribute information model, *BLM* is the behavior logic model and *CRM* is the constraint rule model.

### 3.1. Construction of GPM

The GPM is a 3D descriptive model for physical entities, which is constructed based on the geometric shapes and the assembly and motion relationships of the PEL. The geometric shape model can be realistically rendered, and the relevant assembly and motion relationships are added to the model to ensure consistency in the geometric dimension. In this study, due to the virtual simulation of the FPL focusing on synchronizing the actual production process, the GPM is designed to represent the layout, component positions, and prescribed movements of the PEL. This paper only considers the assembly constraints and motion constraints of the FPL, and does not consider its stress–strain and other continuous physical constraints.

Therefore, the GPM should be regarded as a monitoring-oriented geometric and kinematic model rather than a universal high-fidelity physical simulation model, and the construction process of the GPM designed in this paper can be summarized as follows. When constructing the GPM of the FPL, the hierarchical relationship (“atomic—unit—system” level) is adopted for geometric modeling. For non-functional static components, the model is simplified and only their external geometric shape is retained. For functional dynamic components, the equipment’s own hierarchical structure and motion dependency relationship must be followed during model construction. By establishing the parent–child node relationship between models, corresponding assembly constraints and motion constraints are added to the constructed geometric model. In addition, bounding box-based interference detection can be used for geometric interference-screening operations for selected potentially interacting objects. In this paper, it does not calculate deformation under external loads, gravity-induced structural response, thermal effects, or vibration. Therefore, processes in which these continuous physical fields determine production quality require additional physics-based models and cannot be adequately represented by the present GPM alone.

According to the aforementioned hierarchical structure of the FPL, the GPM can also be divided into the following three models.

#### 3.1.1. Atomic-Level GPM

The atomic-level GPM mainly consists of all the atomic-level equipment and tools that can be independently controlled in the FPL. Among them, atomic-level equipment includes CNC machine tool, industrial robot, automatic guided vehicle (AGV), etc., and atomic-level tools include pallets, cutting tools, rails, the hands of robots, etc. The constructed 3D models of this equipment and these tools have exactly the same dimensions, shapes, and positions as their physical space. At the same time, to further enhance the visual authenticity, these 3D models have also undergone real color rendering.

#### 3.1.2. Unit-Level GPM

The unit-level GPM can be constructed based on the atomic-level GPM and consists of four parts: the atomic-level equipment model, the atomic-level tool model, the material model before entering the production unit, and the product model after the production unit’s processing. According to the composition characteristics of the unit-level in the PEL and the relative positional relationship of atomic-level equipment and tools, the atomic-level GPM is reasonably combined to form the unit-level GPM of a certain production process.

#### 3.1.3. System-Level GPM

The system-level GPM can be constructed based on the unit-level GPM, including the production unit-level models and environmental model of the FPL. According to the relative positional relationship of the unit-level GPM at the PEL, combined with the environmental model, a system-level GPM is formed for subsequent visualization monitoring, which is a relatively complete mapping and description of the production layout of the entire FPL, ensuring that the TML and the PEL maintain a high degree of geometric mirroring.

### 3.2. Construction of AIM

**Figure 2:**
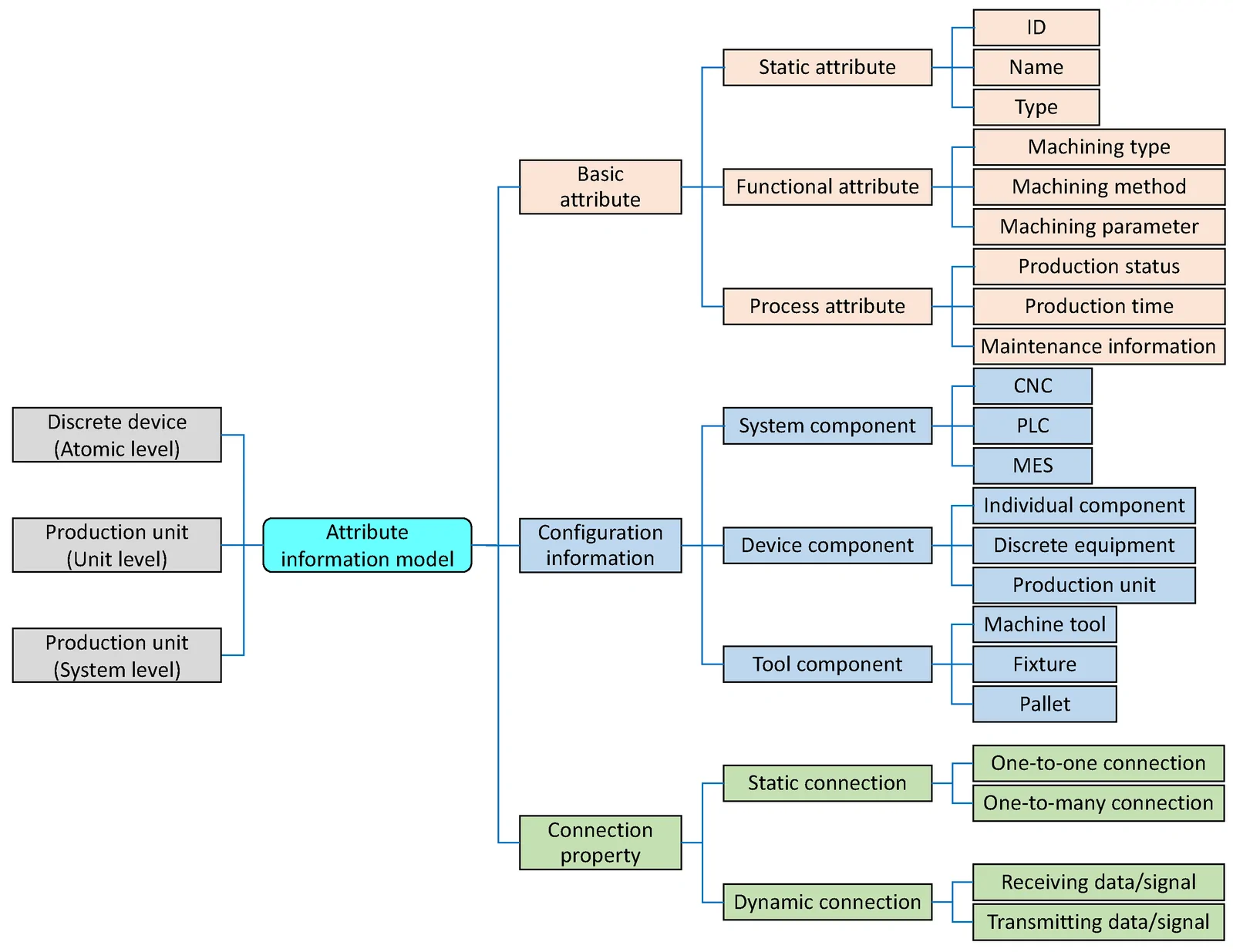
The structure of the AIM for the FLP.

The AIM is used to model the attribute information of the PEL. For an FPL, each discrete device or equipment, each production unit, and the entire production line have their own attribute information. By classifying, defining and expressing the attribute information of the PEL, this relevant attribute information can be assigned to the constructed GPM. Therefore, in an FPL, based on the attribute information of the PEL, these corresponding AIMs can be constructed for the GPM at the atomic level, unit level and system level respectively. Each level’s AIM can describe the model from three aspects: basic attributes, configuration information and connection properties. Its formal expression is as follows:

*AIM* = {*BA*, *CI*, *CP*} (2)

where *BA* = {*BA_i_*|*i* = 1, …, *n*}, *CI* = {*CI_j_*|*j* = 1, …, *m*} and *CP* = {*CP_k_*|*k* = 1, …, *l*} are the set of basic attributes, configuration information, and connection properties, respectively; *n*, *m* and *l* represent the total number of all basic attributes, configuration information, and connection properties, respectively. Thus, the AIM is represented as an ordered triple of three distinct sets, preserving the semantic category of each field. The specific organizational structure of the AIM for the FLP is shown in Figure 2.

#### 3.2.1. Basic Attribute

The basic attributes include a static attribute set, a functional attribute set, and a process attribute set. The static attribute set defines the static attributes of the PEL, such as ID number, name, and type, the functional attribute set defines the functional attributes of the PEL, including machining type, machining method, machining parameters, etc., the process attribute set defines the data related to the production process, such as production status, production time, maintenance information, etc.

#### 3.2.2. Configuration Information

The configuration information is defined based on the configuration of the PEL, and it covers the configuration information at various levels, which is divided into system components, device components, and tool components. The system component describes the composition of systems at each level, such as the CNC and PLC systems at the atomic level, the logistics control system at the unit level, and the manufacturing execution system (MES) at the system level. The device component describes the composition of devices at each level, such as individual components at the atomic level, discrete equipment at the unit level, and a certain unit at the system level; The tool component describes the composition of tools at each level, such as machine tools, fixtures, and pallets, etc.

#### 3.2.3. Connection Property

Connection properties can be divided into static and dynamic connection property. Each level is assigned a unique code to construct its resource and signal I/O attributes. The static connection property refers to the connection between devices in the PEL, including one-to-one and one-to-many connection methods. For example, between AGVs and buffer stations in an FPL, one AGV can correspond to multiple buffer stations, and one buffer station can correspond to multiple AGVs. The dynamic connection property refers to the movement behavior of the model, which is to execute actions based on the signals transmitted by the upper-level system. At the same time, data will also be received during the operation of the equipment itself, that is, data feedback to the upper-level system. For example, after CNC machine tool processing is finished, a completion signal will be transmitted to notify the MES.

### 3.3. Construction of BLM

The BLM describes the dynamic behaviors of objects at the PEL in the FPL. Dynamic behaviors can be divided into motion behaviors of the devices and the logical control of the system for the devices. It mainly involves adding the kinematics and operational logic related to the FPL to the GPM with attribute information, thereby achieving the twin synchronization of the actions or behaviors of the devices within the FPL. Therefore, the BLM consists of two parts: the kinematic model of the devices and the operational logic model of the system. Its formal expression is as follows:

*BLM* = {*DKM*, *OLM*} (3)

where *DKM* = {*DKM_i_*|*i* = 1, …, *p*} and *OLM* = {*OLM_j_*|*j* = 1, …, *q*} are the set of device kinematic model and operational logic model, respectively; *p* and *q* represent the total number of all device kinematic models and all operational logic models of the system, respectively.

For the construction of the device kinematic models, this paper divides them into basic kinematic models and complex kinematic models. The basic kinematic models include the model translation, rotation, and scaling, etc., which can achieve the basic motion control of these models; the complex kinematic models are the models constructed for equipment or tools with complex motion behaviors, such as the interpolation trajectory calculation model for CNC machine cutting tools, the inverse motion solution model for robotic arms, the movement trajectory planning model for AGVs, etc. Currently, there are relatively mature studies on kinematic models and their supporting algorithms. This paper does not provide detailed descriptions here and instead refers to the References [32,33].

For the construction of the operational logic models of the system, this paper utilizes the classic Petri net theory to describe the data flow and production logic of the manufacturing system. Through the object-oriented Petri net modeling method [34], the operational logic of the FPL can be constructed, which can be defined as follows:

*PN*_*OLM* = (*P_i_*, *T_i_*, *R_i_*) (4)

where *PN_OLM* is a Petri net of the operational logic model in the system; *P_i_* is the set of places, which is a finite set indicating the resources and states in the system; *T_i_* is the set of transitions within the system, serving as the transitional transitions connecting *P_i_*; *R_i_* is the set of rule judgments that occur during the transitional transitions.

**Figure 3:**
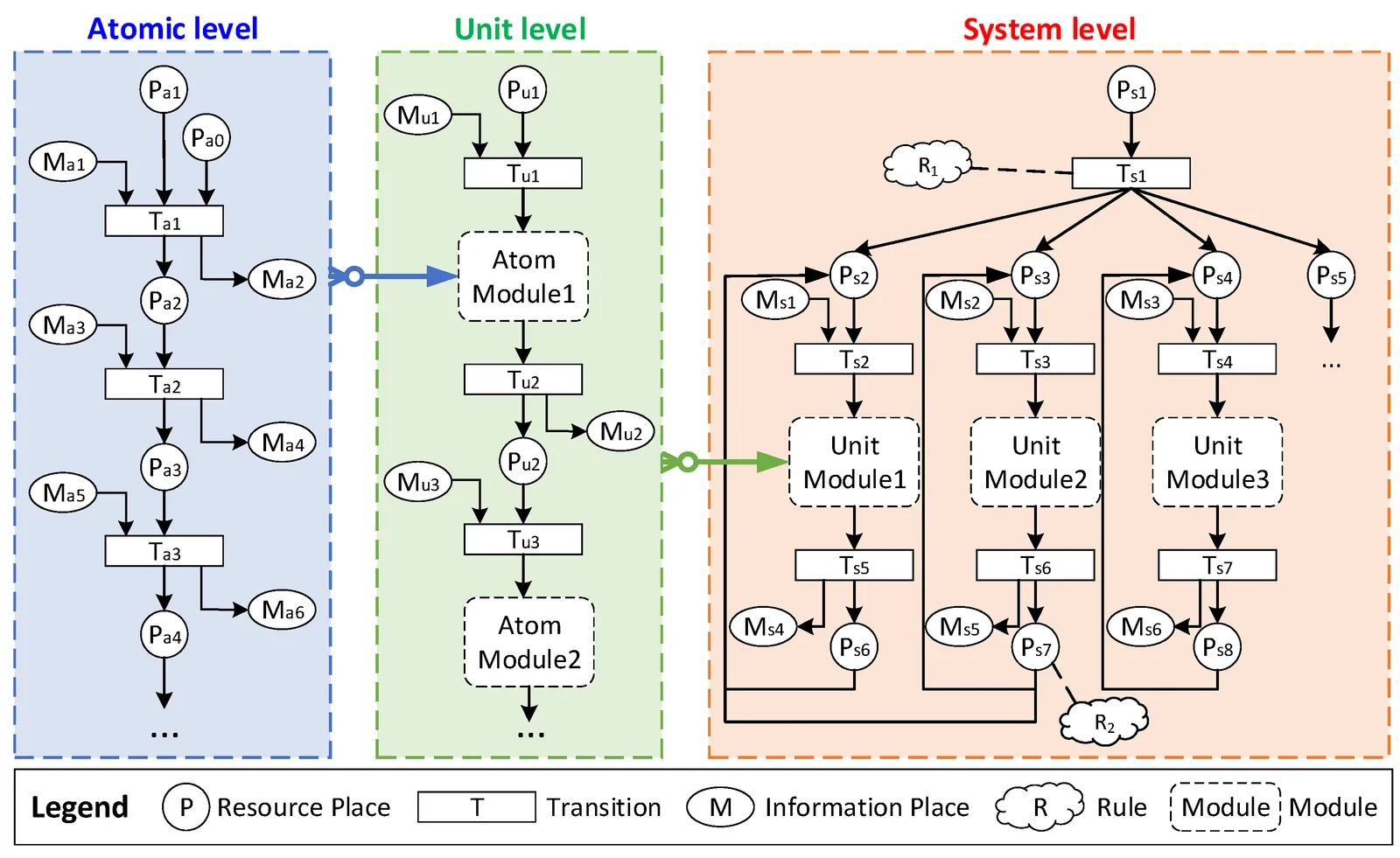
The construction of the operational logic model based on object-oriented Petri net modeling method.

Based on the object-oriented Petri net modeling method, the Petri net of operational logic model of the FPL can be established at the atomic level, unit level, and system level respectively, as shown in Figure 3. Here, *M_i_* represents the information bit required for the transition, divided into the input bit (*M_in_*) and output bit (*M_out_*) of the information.

On the atomic level, as the basic element with motion behavior in the FPL, atomic-level equipment and devices are controlled by sending relevant control commands. The atomic-level operational logical model is constructed based on the fact that the equipment or device receives a signal to complete an action as the basic cell for construction. In the construction of the unit-level operational logic model, the atomic-level operation logic can be encapsulated into blocks (i.e., atom module), and on the unit level, the specific action control of the device is not considered, only the command tasks for the device are sent, and the device executes the processing tasks, and after completion, a corresponding command feedback is given. In the construction of the system-level operational logical model, it is based on the unit level, by issuing tasks to each unit module, allowing each unit-level operational logic model to complete the production tasks of the FPL. Meanwhile, according to the characteristics of the FPL, the behavior of a unit module refers to the logical encapsulation of an individual production unit. Thus, when its dedicated processing function of a certain unit module is unavailable, other production units can continue to operate provided that they do not depend on the unavailable unit. Replacing the unit with a suitable modular production unit and providing its production instructions and behavioral logic are necessary in order to ensure the normal operation of FPL.

Furthermore, to avoid repetitive description and redundancy in the paper, here we will only explain the operation logic of the FPL at the system level, as shown on the right of Figure 3. For example, when the system of a certain FPL receives the production task order (*P_s_*_1_), it can arrange the production task (*T_s_*_1_) based on the production order and characteristics of each production unit, and the production task arrangement follows rule *R*_1_. Each production unit can successively receive the corresponding production tasks. When the production unit module 1 is idle and can receive the production task (*P_s_*_2_), the FPL system sends the production task command (*M_s_*_1_). This production unit receives the task and starts running (*T_s_*_2_), then enters production unit module 1 to perform the task. When the production task is completed (*T_s_*_5_), the task completion command (*M_s_*_4_) is sent. This production unit will become idle again and return to the state, where it can accept a production task order, and so on to complete the entire production task of the FPL. Among them, it should be noted that these relevant rules and their corresponding model construction can be found in Section 3.4 of the following content.

### 3.4. Construction of CRM

The CRM is a representation of the constraints and rules that physical entities of the FPL in the production process need to follow, which is mainly divided into process constraint rule model, operation constraint rule model, production decision rule model, deduction evaluation rule model and knowledge standard rule model. Its formal expression is as follows:

*CRM* = {*PCRM*, *OCRM*, *PDRM*, *DERM*, *KSRM*} (5)

where *PCRM* = {*PCRM_i_*|*i* = 1, …, *r*}, *OCRM* = {*OCRM_j_*|*j* = 1, …, *s*}, *PDRM* = {*PDRM_k_*|*k* = 1, …, *t*}, *DERM* = {*DERM_h_*|*h* = 1, …, *u*}, and *KSRM* = {*KSRM_g_*|*g* = 1, …, *v*} are the set of process constraint rule model, operation constraint rule model, production decision rule model, deduction evaluation rule model, and knowledge standard rule model, respectively; *r*, *s*, *t*, *u* and *v* represent the total number of these above corresponding models, respectively.

#### 3.4.1. Process Constraint Rule Model

**Figure 4:**
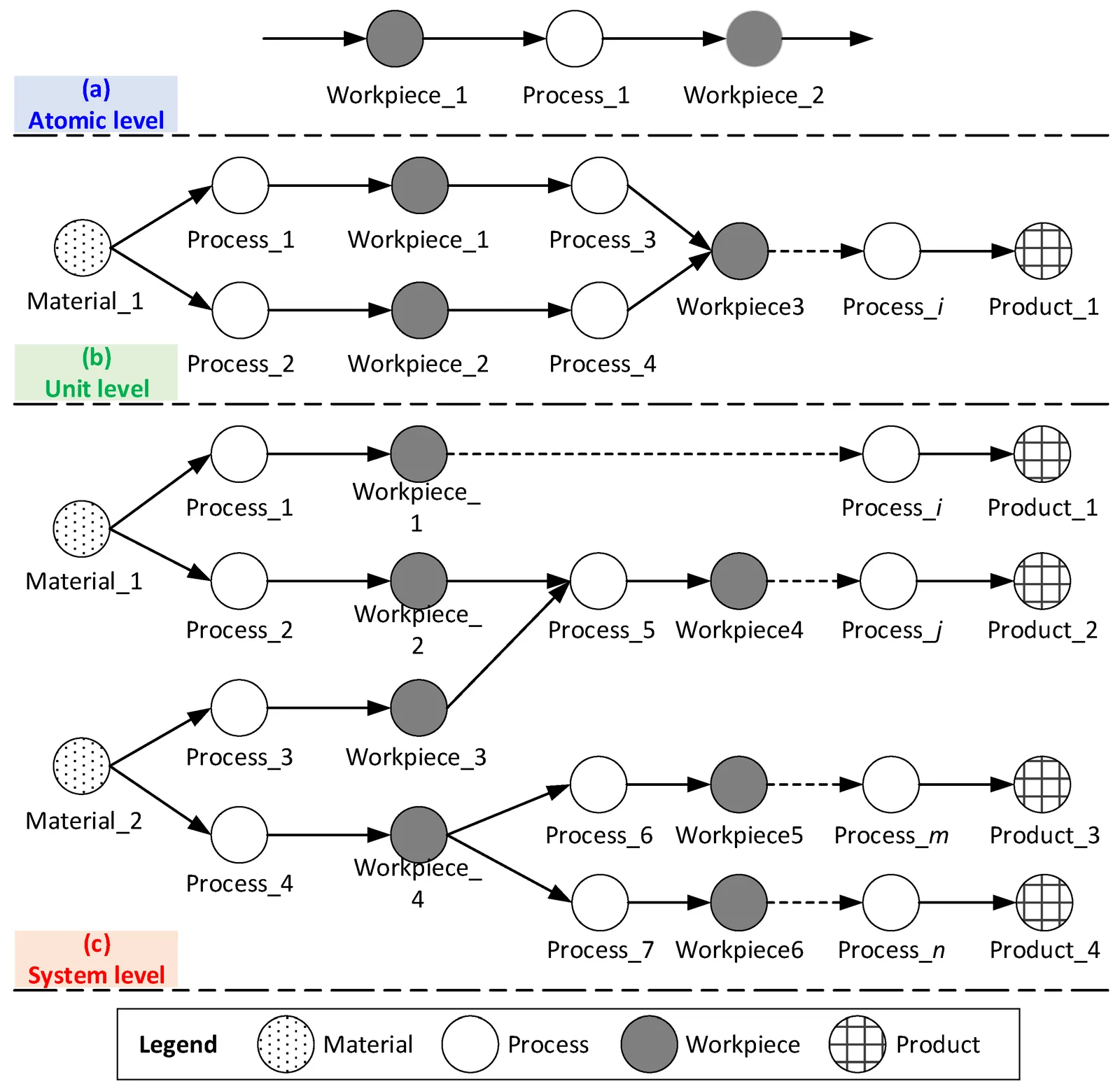
Schematic diagram of the process constraint rule graph constructed using a directed graph approach.

The process constraint rule model describes the production process constraints of the FPL. During the production process, materials need to be machined according to the established production process rules, and eventually become products. Based on the process constraint rules, the accuracy of the processing route in the FPL can be judged, and the evolution rules of materials can also be represented according to the process route. For example, the working process model of the product is determined by its process constraint rules. When constructing the process constraint rules, the model can be constructed respectively at the atomic level, unit level, and system level using a directed graph approach, as shown in Figure 4.

#### 3.4.2. Operation Constraint Rule Model

**Figure 5:**
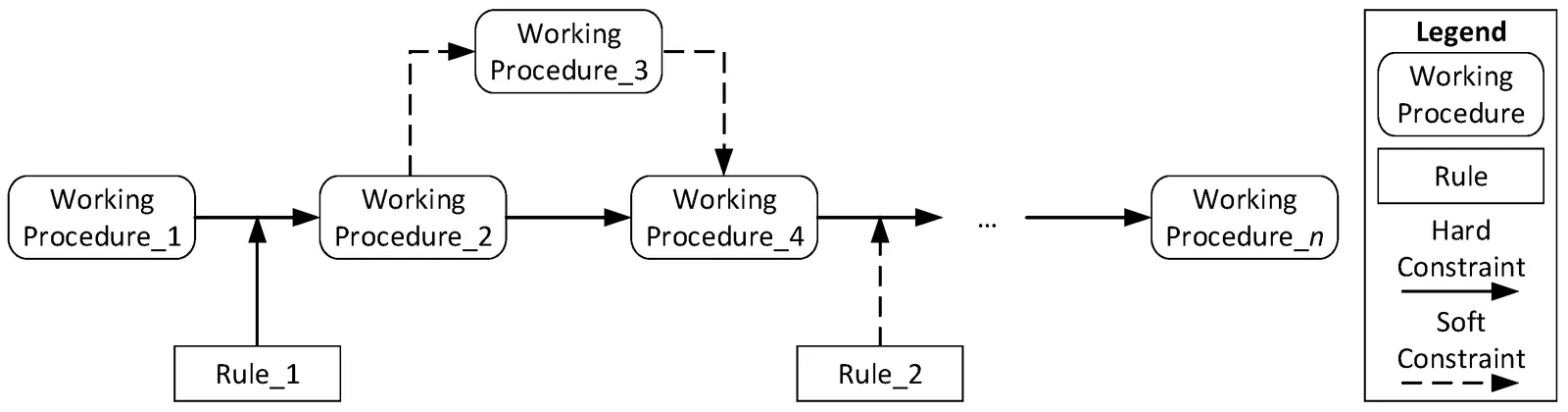
Schematic diagram of the operation constraint rule model.

The operation constraint rule model is used to construct the relevant constraint rules in the production process of the FPL. Based on the characteristics of operation constraints, they can be classified into hard constraints and soft constraints. Hard constraints refer to the rules that must be followed in the production process; soft constraints refer to the rules that can be selectively executed in the production process according to the actual production status on site or the scheduling arrangement of upper-level instructions. Similarly, the construction of the operation constraint rule model can also be implemented from the perspectives of the atomic level, unit level, and system level respectively. The specific manifestations of the operation constraint rule are shown in Figure 5.

#### 3.4.3. Production Decision Rule Model

**Figure 6:**
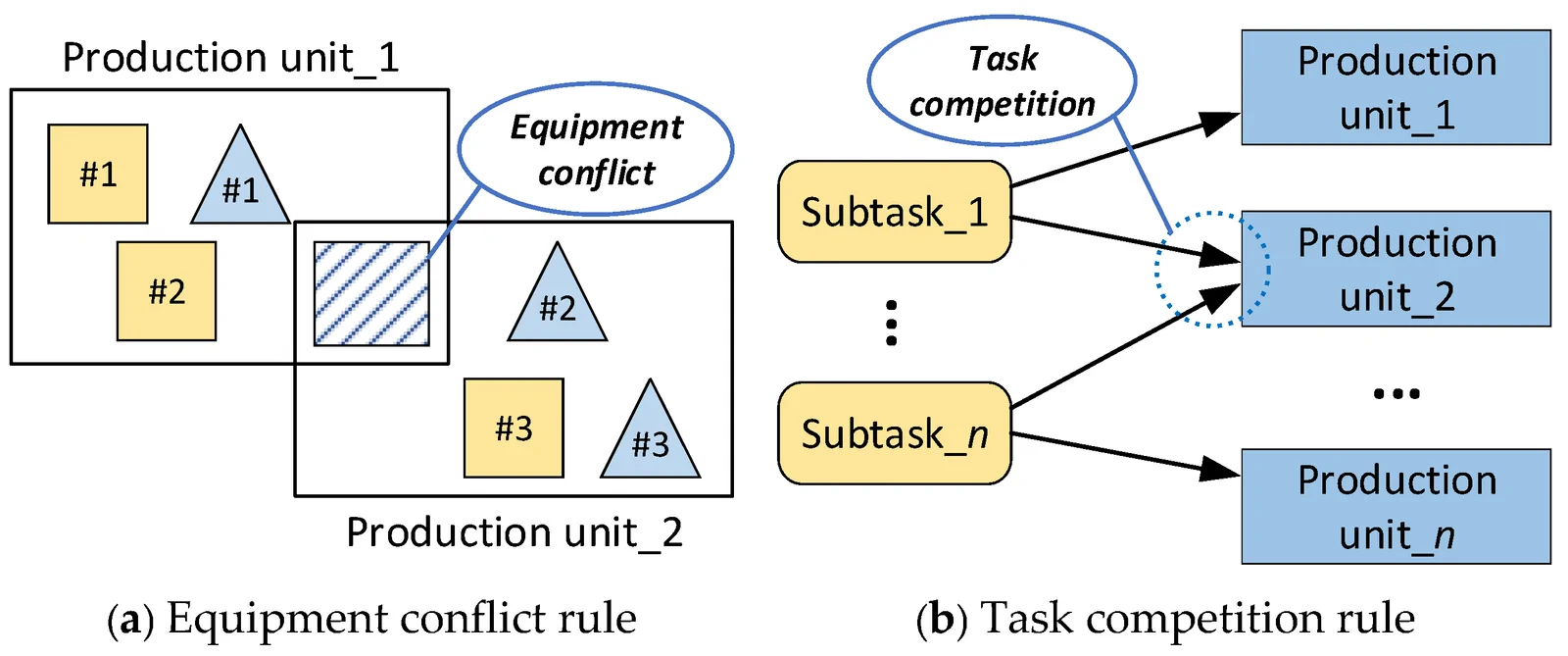
Schematic diagram of two typical production decision rules.

The production decision rule model defines the judgment and decision-making rules during the production process of FPL. During the production process, when all relevant conditions are met, production decision rules are needed to assist in making judgments. Typical production decision rules include equipment conflict rules and task competition rules, as shown in Figure 6. Equipment conflict occurs when different production units of an FPL share the same equipment. The shared equipment may receive two different tasks at the same time, so equipment conflict rules need to be established to make judgments and determine the priority of tasks; while task competition occurs when a single production task can be completed by multiple production units, so task competition rules need to be established to determine which production unit will complete the production task.

#### 3.4.4. Deduction Evaluation Rule Model

Based on the historical operation process of the FPL, the deduction evaluation rule model can be constructed by the relevant mathematical models to predict and assess the actions, behaviors, and states of the actual production process, including production status evaluation, product quality evaluation, and task scheduling rationality evaluation. Therefore, during the production process of the FPL, the real-time data-driven operation results can be displayed to users through deduction evaluation rules, providing guidance for the actual production. For example, the machining time of products can be predicted based on historical machining data and the current status, and the preventive maintenance cycle of the CNC machine tool can be determined using the maximum available working degree method.

#### 3.4.5. Knowledge Standard Rule Model

The knowledge standard rule model can be established based on historical experience, production knowledge, and industry standards of the FPL. Among them, production knowledge includes equipment maintenance experience, FPL commissioning experience, and methods for handling alarms; industry standards include equipment communication standards and FPL manufacturing standards. The above knowledge standard rules can play an important role in the daily operation and maintenance of the FPL, reducing unnecessary downtime and failure time.

## 4. Visualization Monitoring of Flexible Production Lines Driven by Real-Time Data

### 4.1. Data Interconnection Mechanism in the FPL

**Figure 7:**
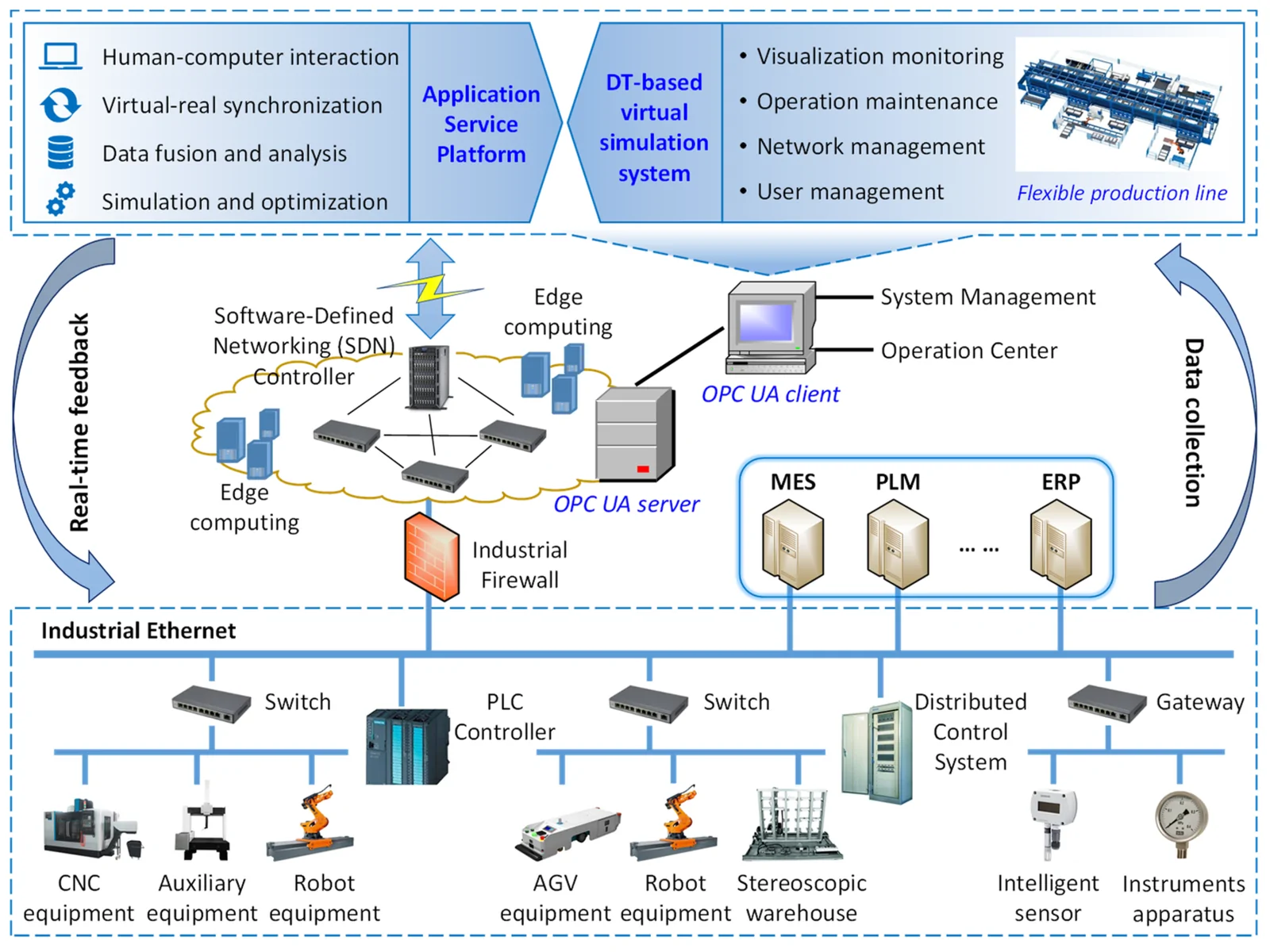
Schematic diagram of the CTL construction through the data interconnection mechanism.

In the process of constructing the CTL of the aforementioned digital twin framework, the establishment of the data interconnection mechanism is one of the basic requirements for achieving visualization monitoring of the FPL. Figure 7 is a schematic diagram of the CTL construction through the data interconnection mechanism. Among them, the connection and transmission methods include: (1) data collection and feedback at the PEL, (2) data reading and storage at the database, and (3) data sharing and storage among different application software.

The data collection and feedback at the PEL are the most crucial part of the FPL. To achieve visualization monitoring of the FPL, it is essential to obtain a large amount of real-time data during the production process and ensure high-quality transmission. By leveraging the data interconnection mechanism, communication connections can be established among different types of systems or devices in the PEL based on network communication protocols. Through the data dictionaries and operation specifications of the equipment, consistent parsing of real-time data can be achieved. It should be noted that the current common data collection method involves system development based on the OPC UA protocol and upper computer development based on the software development kit (SDK).

The data reading and storage in the database are based on the selected database type. Using the Structured Query Language (SQL), operations such as adding, deleting, modifying, and querying data are carried out according to the interface of the corresponding database.

The data sharing and storage among different application software are developed based on the IT data transmission protocol. When developing related applications, it is necessary to call the relevant data according to the application programming interface (API) defined by the developers, so that the data can be shared and stored among different application software.

Through the proposed data interconnection mechanism and corresponding connection and transmission methods, it is possible to achieve data collection, analysis and storage of production information such as the machining status, numerical control, sensor data, and so on. The paper is based on the OPC UA data communication network architecture to realize data collection of the PEL in the FPL. This is mainly done by embedding the OPC UA server into various production devices (such as CNC machine tools, industrial robots, etc.), PLC, and production management systems (such as MES) of the FPL. According to the data dictionaries of each device or system, these corresponding information models can be constructed, thereby enabling real-time data collection of production information and control instructions from these devices or systems. In addition, these edge computing devices are set up for real-time processing and analysis of feedback signals from various types of sensors. These feedback signals can reflect the status information of production devices, such as health status, motion status and so on. Therefore, the established data interconnection mechanism can ensure high-speed data transmission when connecting various devices or systems at the PEL of the FPL, and can further guarantee the timeliness of the production data.

### 4.2. Twin Model Mapping Driven by Real-Time Data in the FPL

In order to utilize the real-time data collected at the production line for online driving and dynamic mapping of the twin model, it is necessary to classify and process the real-time data collected and transmitted at the production site. This paper classifies it into four types of real-time data in the FPLs: production status data, production control data, production process and rule data, and production alarm data. Among them, production status data are used to drive the AIM of the TML, mainly adjusting and updating the attribute information of the twin model based on the obtained production line status data; production control data are used to drive the BLM of the TML, which includes equipment motion data and control instruction data. Through the control instruction data based on the behavior logic, the equipment can be controlled, and then the model position of the TML can be updated and displayed according to the equipment motion data; production process and rule data are used to drive the CRM of the TML, which includes machining process data, process requirement data, measurement data, etc., generated during the manufacturing process of the FPL. When implementing the dynamic mapping of the twin model based on process and rule data, by comparing the results driven on real-time process data and those related constraint rule data, the production operation process of the FPL can be further evaluated and optimized; production alarm data are used to accurately locate alarm errors and other reasons in the manufacturing process of the FPL, reducing the time spent by maintenance personnel on troubleshooting faults and errors of the production line.

**Figure 8:**
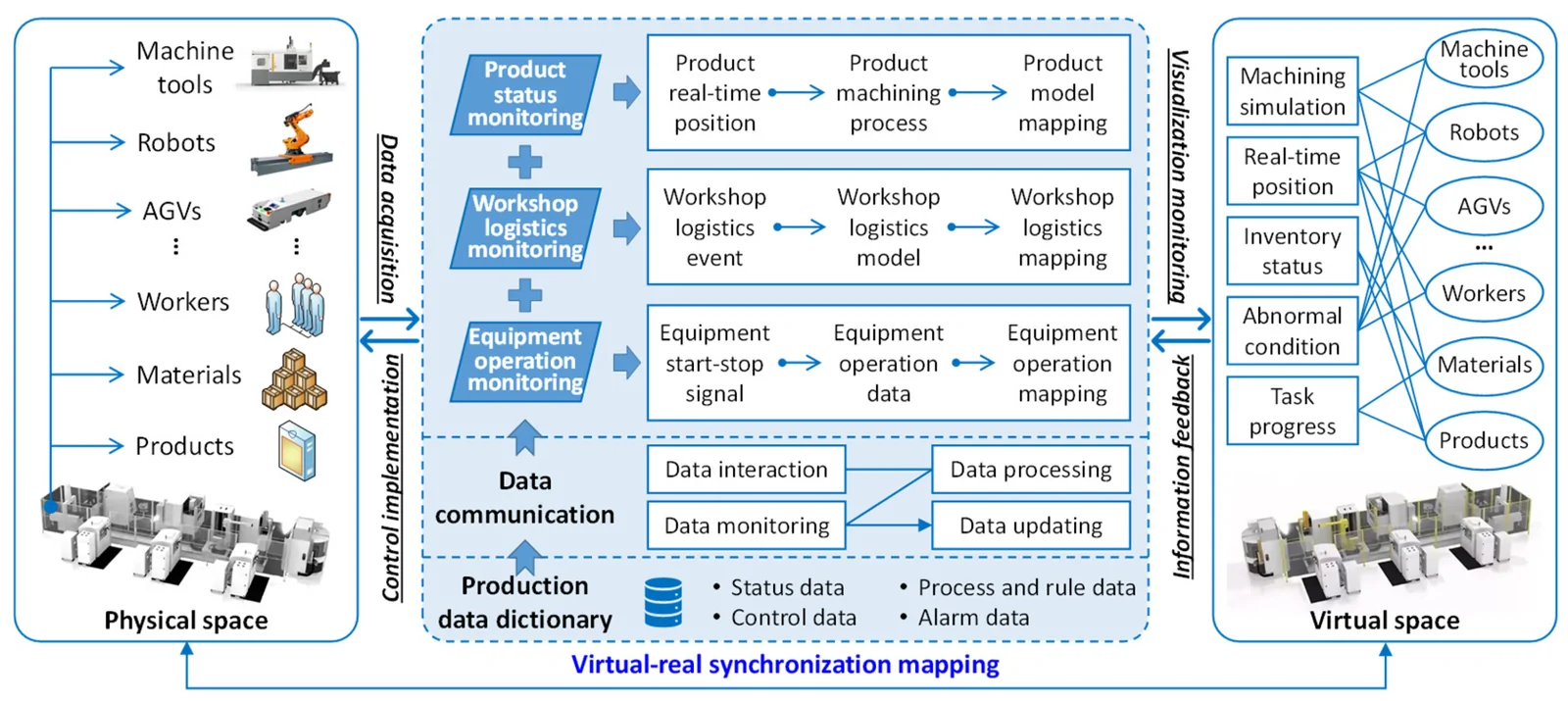
Schematic diagram of the virtual-real synchronization mapping of twin model in the FPLs.

Due to the complex, multi-source, and heterogeneous nature of the real-time data in the PEL of the FPLs, in order to achieve online driving and dynamic mapping of the twin model, a unified data dictionary has been constructed from the collected real-time data for the visualization monitoring of the FPLs, which describes the data structure, data items, data control logic, and data flow in it. This enables consistent parsing of data interconnection between the PEL and the TML. With the support of real-time data-driven virtual-real synchronization mapping, the online and dynamic operation process of the FPL can be visualized and displayed, achieving 3D visualization monitoring of the entire process of the FPL, as shown in Figure 8. Here, taking the visualization monitoring of CNC machine tool protective door as an example, we will explain the specific implementation process of the twin model mapping driven by real-time data in the FPL. When the physical CNC machine tool protective door changes from the closed state to the open state, the corresponding digital signal can be acquired from the production control system through the data acquisition interface. The signal is timestamped and parsed according to the data dictionary of the CNC machine tool. It is then transmitted through the CTL to the TDL, where the door-state attribute of the corresponding twin model of CNC machine tool is updated. The updated door-state is simultaneously evaluated against the relevant process constraint rule of the CRM. If the operation of CNC machine tool requires the protective door to remain closed during a particular machining stage, the process constraint rule model can identify the door-state as an abnormal process condition. The ASL will then generate a warning message and highlight the corresponding twin model of CNC machine tool protective door in the 3D visualization monitoring interface. In addition, when a control action of CNC machine tool protective door is required and supported by the production control logic, the closing-door command can be modified, and the PLC configuration can be reset through the editor of the developed DT system. The corresponding command can be returned to the PEL through the CTL until the closing action of the CNC machine tool protective door is completed. Meanwhile, the position sensor on the protective door will detect whether the door is fully closed and send the signal back to the PLC, ensuring that the CNC machine tool cannot start processing until the protective door is properly closed.

Furthermore, since the real-time data collected on the FPL is prone to errors such as null values, repetitions, and anomalies, it is necessary to perform operations such as interpolation, filling in blanks, and removal of duplicates from these data. In order to improve the preprocessing quality of the real-time data, the data operations should be further specified. For continuous numerical variables, missing samples can be reconstructed using common methods for handling missing data, such as forward filling, moving average, exponential smoothing, linear interpolation, etc. For discrete state variables, the most recent valid state is retained until a new valid state is received. Duplicate records can be identified according to the equipment identifier, timestamp, and data-item identifier, and repeated records are removed. In addition, for variables with predefined engineering limits in the manufacturing process of the FPL, values outside the valid range should be marked as abnormal and excluded from twin model mapping. On this basis, through establishing the corresponding data parsing algorithm models, the pre-processing and cleaning of the collected real-time data on the FPL can be achieved, thereby completing the effective transmission of real-time data in the PEL and supporting the subsequent visualization monitoring service of the production line.

### 4.3. Twin Data Management for Visualization Monitoring of FPL

As the support for the DT-based virtual simulation system for visualization monitoring of FPLs, the TDL serves as the operational foundation for the TML and the ASL. The reasonable composition and efficient management of twin data can determine the level of visualization monitoring for FPLs.

Twin data are the core driving source for visualization monitoring of FPLs. The pre-processed twin data can be stored in the data dictionary, and the data queue can be updated through the periodic data flows, thereby providing conditions for visualization monitoring of FPLs. For the twin data required for visualization monitoring, a twin data management model is proposed and a periodic data update template is established, which drives the operation of the twin model and enables the traceability of the production process. Based on the composition components of the TDL, the twin data can be classified into physical entity data, twin model data, application service data, and the integrated data among the three, as described in detail below:

(1) Physical entity data mainly refer to the data collected during the dynamic production process of the FPL through sensors, data acquisition systems, etc., which are divided into production status data and production control data. Production status data refer to the real-time status data during the production operation of the FPL, including the machining status data of equipment and tools, as well as the manufacturing status data of materials and products, such as the usage status data of tools and fixtures, and the production quality or inspection result data of machined parts, etc. Production control data refer to the data related to the logical control of production tasks allocation and scheduling, operation management, and processing logistics during the production operation of the FPL.
(2) Twin model data mainly include basic attribute data and virtual simulation data. Among them, basic attribute data includes production static data, production resource data, alarm and maintenance data, etc., virtual simulation data are the data simulated in the TML based on physical entity data, which includes data for analysis, verification, prediction, and optimization during the virtual simulation process of the FPL.
(3) Application service data mainly consists of application analysis data and knowledge service data. Among them, application analysis data are the data obtained through the statistical analysis of physical entity data and twin model data, which is developed by the relevant application services based on the visualization monitoring tasks of the FPL, such as real-time status data of production equipment, task progress data of products in process, and workshop abnormal data; knowledge service data refer to the data providing relevant knowledge services for the production process of the FPL, such as historical experience data of workers and safety standard data of production.
(4) Fusion derived data are mainly the fused data and derived data obtained by pre-processing, classification, association, and integration methods from the corresponding data generated by the PEL, TML, and ASL, which can provide further value-added services for the visualization monitoring of the FPL. For example, based on the real-time position of the workpiece and process flow data in the PEL, the current production process being carried out on the production line can be determined after data processing and analysis.

**Figure 9:**
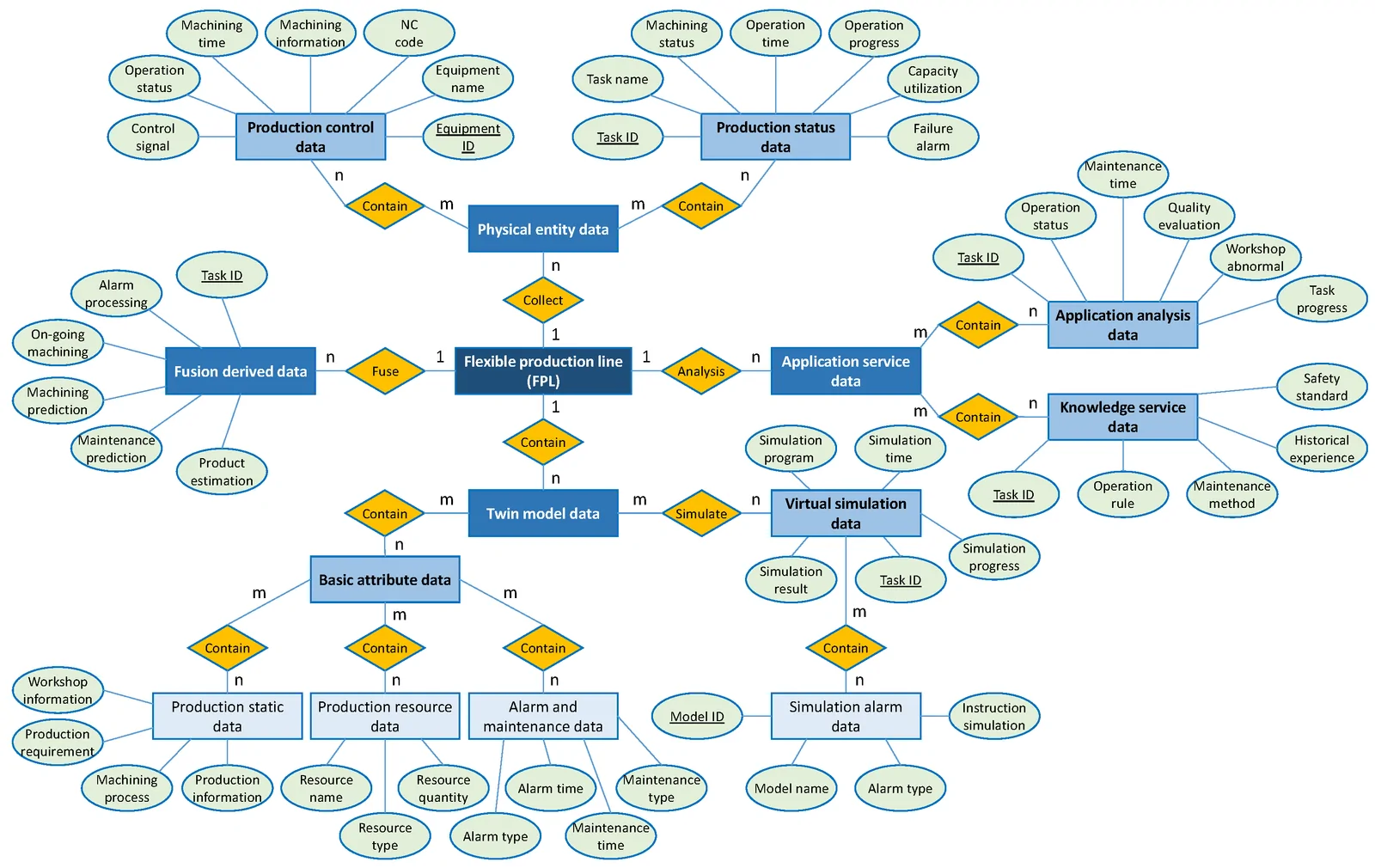
An E-R diagram of the twin data for visualization monitoring of FPLs.

Considering that the composition of the twin data for visualization monitoring of the FPLs is rather complex, this paper manages the twin data by constructing a database. According to the management requirements of the twin data, an E-R diagram is adopted to construct the entity sets of each data type, as well as their corresponding attributes and interrelationships, as shown in Figure 9. This can form a complete data chain for each data type, thereby completing the storage and management of the twin data.

## 5. Case Study

**Figure 10:**
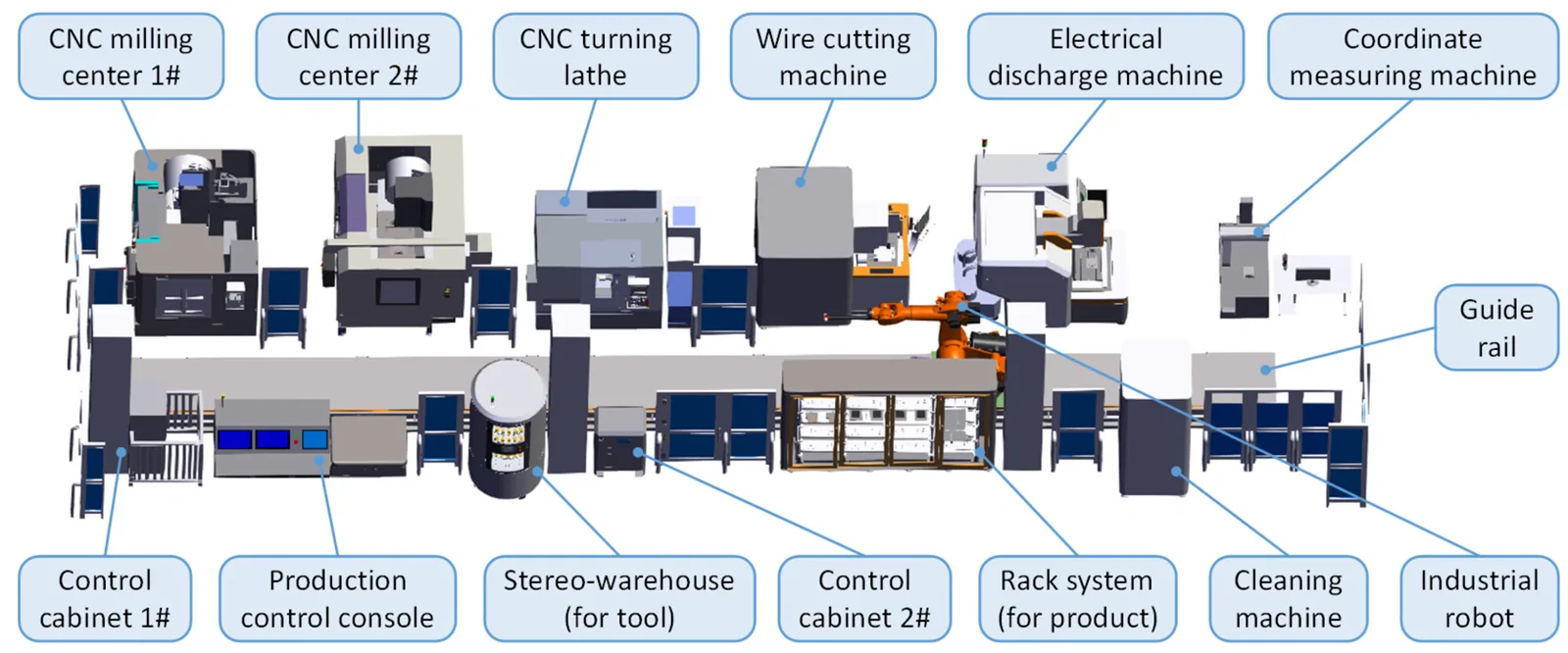
Production layout diagram of a typical FPL in a certain machining scenario.

In order to develop and verify the application of a virtual simulation system under the digital twin framework for visualization monitoring of FPLs, this paper selects a typical FPL in a certain machining scenario as the research example, as shown in Figure 10. The FPL integrates CNC milling center, CNC turning lathe, wire cutting machine, electrical discharge machine, coordinate measuring machine (CMM), cleaning machines, industrial robot, and automation stereo-warehouse/rack system, etc., which has functional modules such as numerical control and special processing, positioning, detection, cleaning, intelligent logistics and warehousing, and control. The FPL can realize processes such as CNC milling, CNC turning, slow wire cutting, and electrical discharge machining. The industrial robot moves along the guide rails to complete the material transportation and loading/unloading tasks between various workstations of the FPL, and the entire production process can be scheduled and automated through the manufacturing execution system (MES) and programmable logic controller (PLC) system.

**Figure 11:**
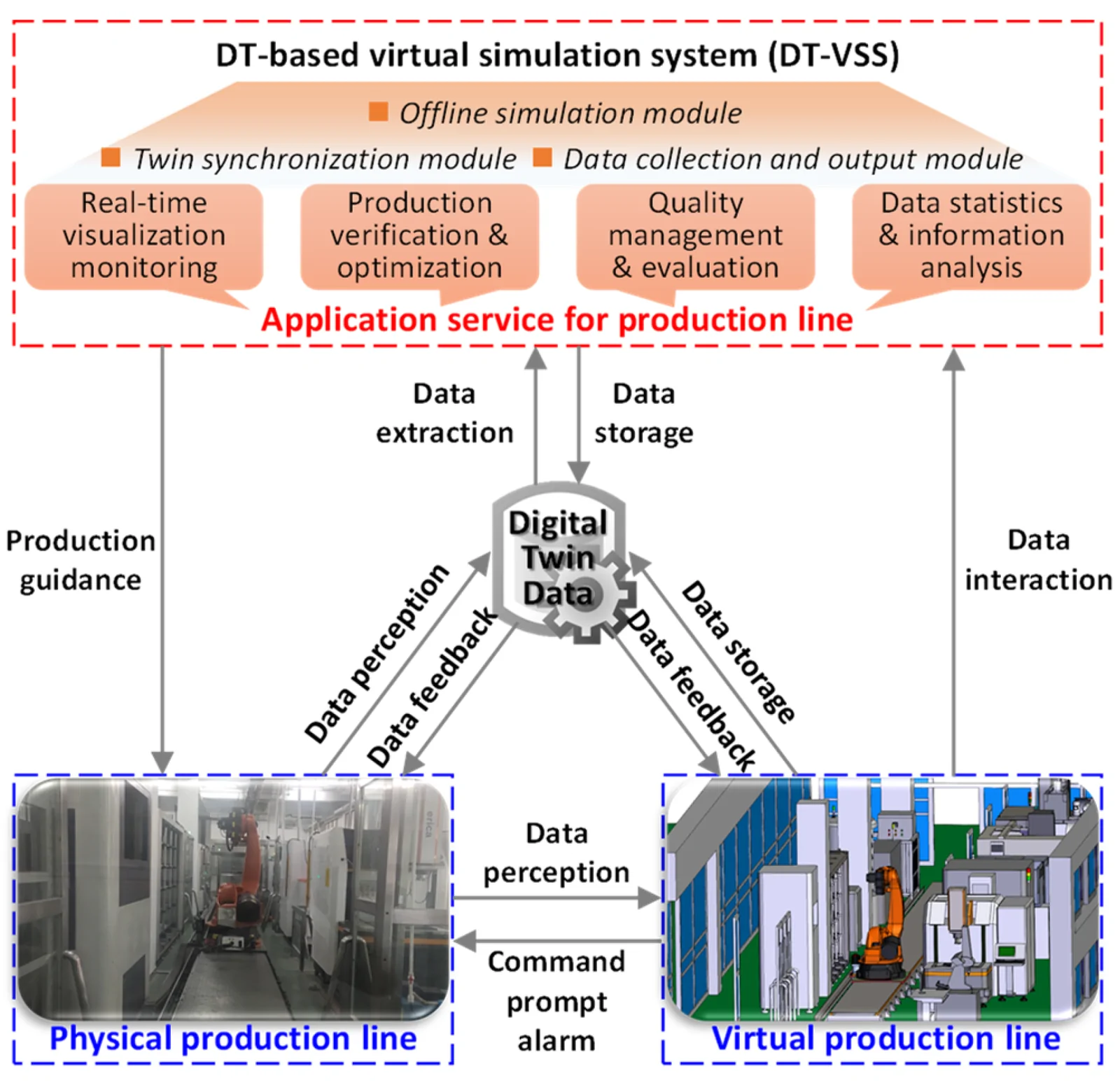
Schematic diagram of the DT-VSS construction for 3D visualization monitoring of FPL.

In response to the operational requirements of the FPL for safety, efficiency, and visualization monitoring, this paper has developed a prototype DT-based virtual simulation system (DT-VSS) for the FPL. The DT-VSS is based on the Browser/Server (B/S) architecture. The front-end development uses HTML5, CSS3 and JavaScript to create the web interface, and the 3D model visualization of the FPL is realized based on the WebGL technology. The back-end development is mainly responsible for data collection, processing and feedback control of the PEL, which can be implemented through the Mitsubishi^®^ MX component and realized using the C# language based on the .NET framework. To facilitate the reading of the 3D model data of FPL, the PostgreSQL database is used for data storage, and finally, through the MVC (Model—View—Controller) programming mode under the ASP.NET development framework, the integration of the entire DT-VSS can be realized. The real-time data transmission for the FPL can be achieved through the HTTP protocol and WebSocket technology. The schematic diagram of the DT-VSS construction for 3D visualization monitoring of FPL is shown in Figure 11. In the DT-VSS, the real-time data transmission and 3D rendering of the FPL are treated as independent but coordinated processes. WebSocket is responsible for asynchronous transmission of updated twin data, while the WebGL engine of the front-end development maintains the 3D scene and updates only the twin models whose states have changed. Static geometric models of the FPL can be loaded and retained in the client, so the arrival of a new twin data packet does not require reconstruction of the entire scene of the FPL. Meanwhile, in order to enhance the efficiency of computer graphics processing, it is necessary to reduce the memory occupied by the static geometric models through lightweight processing during the 3D modeling process. The lightweight processing is mainly achieved by retaining the key components and simplifying those components that are unrelated to the graphical twin model. Consequently, the computational load of real-time synchronization mainly depends on the number of changed models and attributes rather than the total complexity of the 3D model in the 3D visualization monitoring of the FPL.

**Figure 12:**
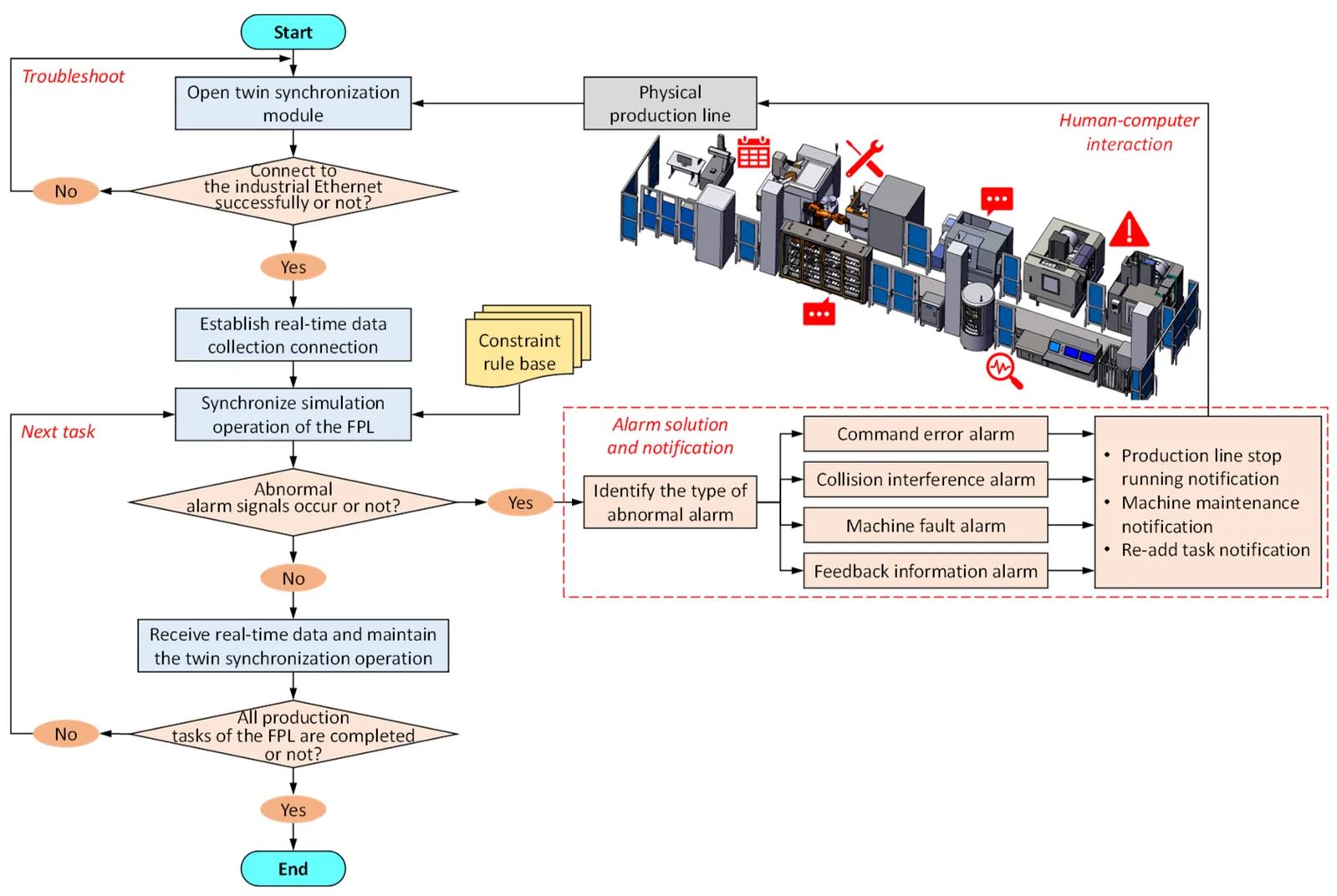
Flow diagram of the twin synchronization module for virtual-real synchronization interaction.

In the DT-VSS, each twin model is a mirror mapping of the actual FPL, and its production environment and layout are also consistent with those of the PEL in the actual production line. The DT-VSS consists of three modules: offline simulation module, twin synchronization module, and data collection and output module. Among them, the most core functional module is the twin synchronization module, which mainly realizes the real-time visualization monitoring of the FPL through twin synchronization and virtual-real interaction with the physical production line. In the twin synchronization module, twin data includes not only the real-time generated processing data but also various interactive feedback data and real-time control data during the execution process of the FPL. The DT-VSS can utilize these twin data to achieve virtual-real synchronization with the physical production line, and the virtual-real synchronization interaction process is shown in Figure 12. The DT-VSS will first conduct simulation and verification in the offline simulation module based on the machining process file. Once confirmed to be error-free, it will proceed with the actual production operation and activate the twin synchronization module for real-time visualization monitoring. The subsequent specific steps are as follows:

Step 1: Open the twin synchronization module and connect to the industrial Ethernet, and activate the data acquisition and output module for real-time connection. Once the connection is confirmed to be successful, proceed to the next step. If any related alarm errors occur, the user needs to troubleshoot the errors until the connection is successful without any errors;

Step 2: Click to start the twin synchronous simulation operation, and simultaneously start the production operation of the FPL. The DT-VSS will perform the twin synchronization operation of the FPL based on the twin data. If abnormal alarm signals occur during the twin synchronization operation, identify the type of abnormal alarm, click the corresponding button according to the prompt, and provide the corresponding solution until the abnormal alarm signal is eliminated, and then proceed to the next step;

Step 3: The DT-VSS continues to receive real-time twin data and maintains the twin synchronization operation until all production tasks of the production line are completed.

**Figure 13:**
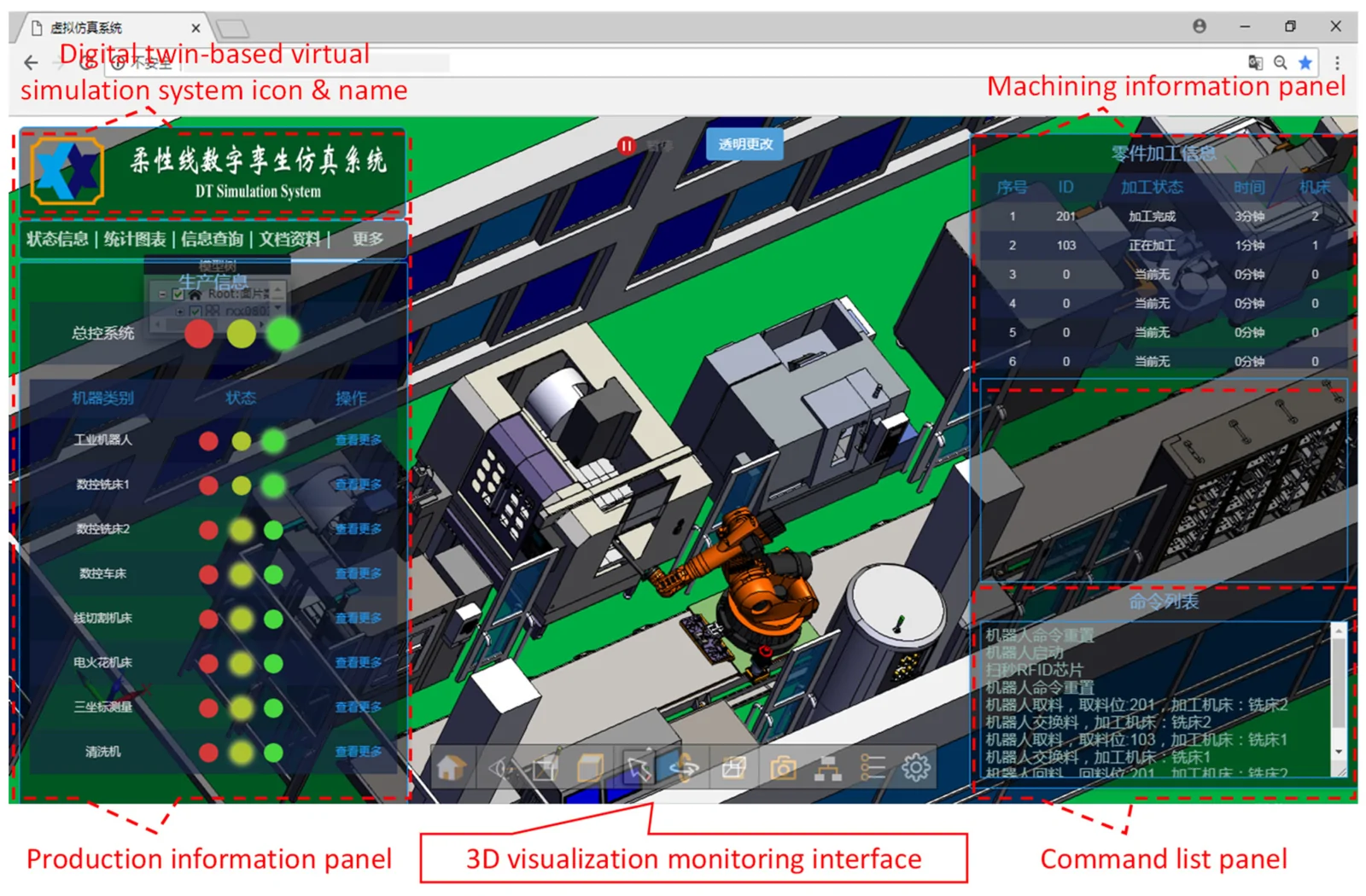
3D visualization monitoring interface and panels of DT-VSS.

**Figure 14:**
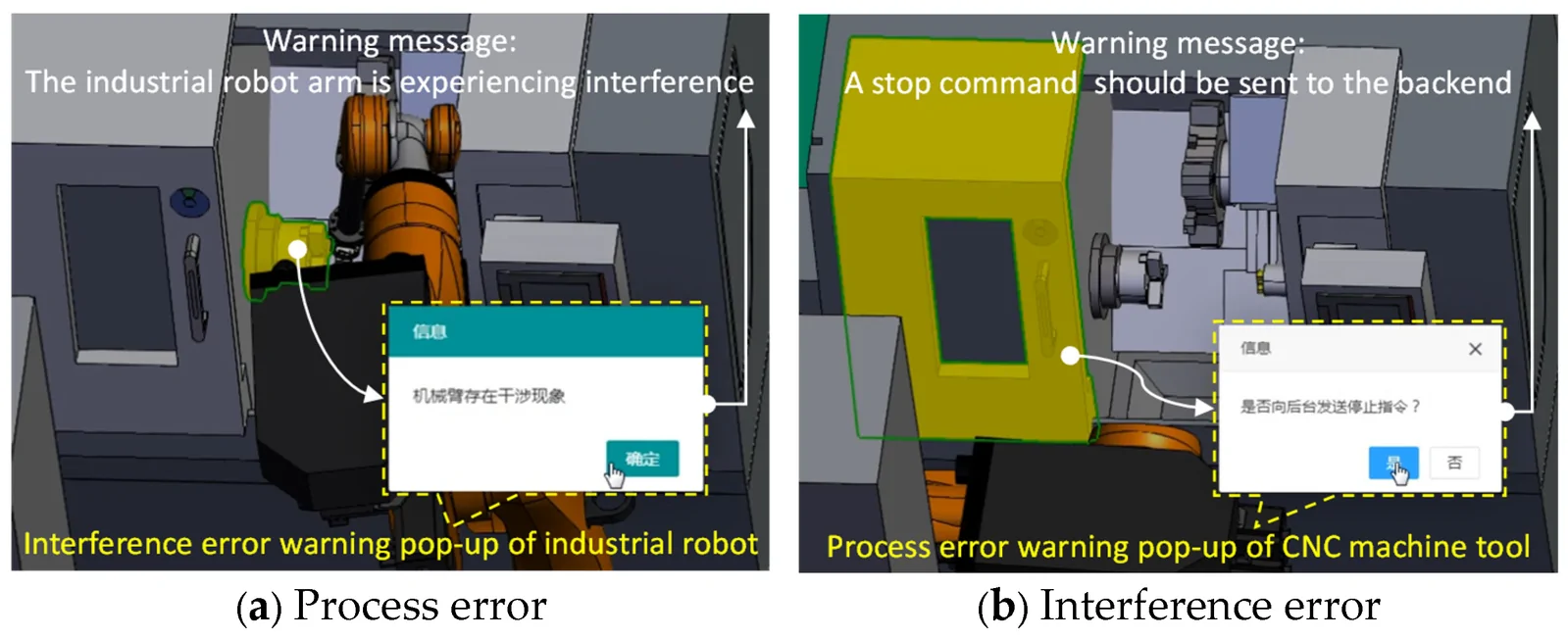
Warning pop-ups of abnormal alarm signal of the FPL in the DT-VSS.

During the operation of the DT-VSS, the user can use human–computer interaction to display or hide the status information panels of each device and workpiece, and the comprehensive statistical information will be retained in the display interface, as shown in Figure 13. Meanwhile, the related data analysis messages (such as abnormal alarm signals) of the production line will be alerted to the workers in the form of warning pop-ups. For example, Figure 14a shows a warning pop-up when a process error happens during the operation of production line, which is triggered due to the failure to close the CNC machine tool protective door promptly after removing the machined workpiece. The above process does not conform to the process rule set by the DT-VSS, causing the system to issue a warning pop-up and highlight the corresponding model (i.e., CNC machine tool protective door). Figure 14b shows a warning pop-up when an interference happens during the virtual simulation of production line. This warning mainly alerts the user when there is a collision and interference during the motion simulation of the twin model of industrial robot. The DT-VSS will immediately feed back the alarm signal to the PEL, causing the interfering equipment to stop moving. It should be noted that for the feedback control of physical devices, the DT-VSS can connect to the related controller through Mitsubishi^®^ MX component. Based on human–computer interaction, the user can use the editor of the DT-VSS to modify the PLC, thereby achieving feedback control of the physical devices by reconfiguring the settings.

**Figure 15:**
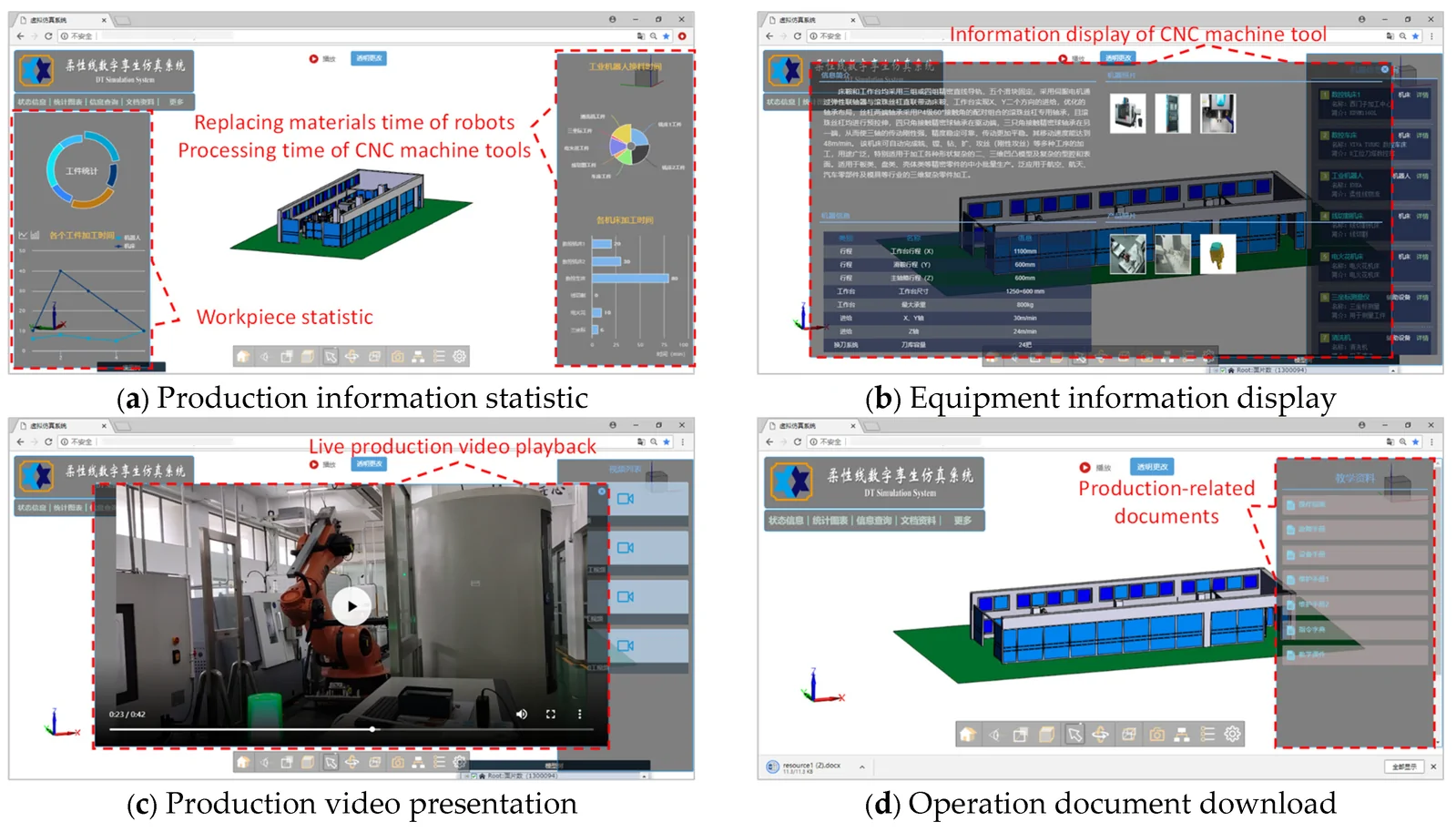
Display interface of different auxiliary function modules in the DT-VSS.

In addition to the 3D visualization monitoring interface, the DT-VSS also builds different auxiliary function modules such as production information statistic analysis, equipment information display, production video presentation, operation document download, etc., as shown in Figure 15, which can monitor aspects like machining process, working hours, processing progress, and resource status, and present the data of production line in detail through the format of text and chart.

Through the on-site implementation and application of the DT-VSS, the twin synchronization and real-time 3D visualization monitoring of the actual FPL have been basically achieved. These functions of monitoring the operation status, tracking product materials, data statistical analysis, and alarm information feedback in the FPL have been completed, which has enhanced the 3D visualization monitoring capabilities of the FPL and ensured the smooth and safe operation of the entire production process. It is necessary to specifically point out that when conducting the on-site testing of the DT-VSS for the representative machining FPL, in order to balance the real-time monitoring requirements and the system communication load, we set the polling period through the Mitsubishi^®^ communication plugin to 200 ms. That is, the frequency of on-site data collection and feedback are updated once every 200 ms, ensuring that the ASL of the FPL can obtain a snapshot of the on-site status at a frequency of 5 Hz, which is sufficient to cope with the monitoring of most equipment status and process parameters. However, for rapid dynamic processes that require microsecond/millisecond-level response, this may constitute an observation bottleneck. Finally, these results indicate that the developed DT-VSS can provide a technical solution for achieving the virtual-physical mapping and 3D visualization monitoring of the entire production process of the FPL. This also suggests that the task-action mapping of the FPL has an acceptable fast response speed and high consistency.

## 6. Conclusions and Future Work

This paper is aimed at the intelligent development needs of FPLs in the production workshop and the problem of 3D visualization monitoring of FPLs. It proposes a five-layer DT framework for 3D visualization monitoring of FPLs, analyzes the constituent elements of each level of the framework structure, and on this basis, designs the twin model of the FPL at three levels (i.e., atomic level, unit level, and system level). It presents the construction methods and implementation paths of the twin model of the FPL from four dimensions: geometry physics, attribute information, behavior logic, and constraint rule. Furthermore, it also studies the real-time data-driven visualization monitoring method for FPLs, analyzing the key technologies involved in three aspects: data interconnection mechanism, twin model mapping, and twin data management methods. Finally, a prototype DT-VSS for the FPL is developed, and a typical FPL in a machining scenario is used as an example for on-site practical application, verifying the effectiveness and feasibility of the DT-VSS and achieving the 3D visualization monitoring of the machining process.

The preliminary research and exploration in the application of real-time visualization monitoring of FPLs have been conducted through the construction method of DTS and its five-dimensional DT model, achieving the visualization monitoring tasks such as the current operation status and historical status of the FPL. Although the research results of this paper have been applied to a certain machining FPL and have achieved certain effects, there are still the following limitations. First, due to the fact that some of the equipment is from foreign manufacturers, the data collection process is quite challenging and the real-time production data has not yet been fully collected. Second, validation was conducted in a single machining production scenario and covered selected monitored objects and events, rather than providing verified coverage of every object and every process throughout the line or shop-floor. Third, this study did not include a baseline comparison and a sufficiently statistical dataset, which lacks a comparative assessment for the visualization monitoring of the entire production process. The future research works will focus on integrating analysis and value-added services for the operation process of the FPL and using more actual physical constraint conditions and real-time perception data to predict future operation trends, abnormalities and bottleneck resources, and optimize the production process and production resource allocation of the FPL, thereby feeding back to the physical production line and establishing a more truly closed and highly mature DTS, so as to promote the intelligent development of the FPL.

## Data Availability

The data that support the findings of this study are available from the corresponding author upon reasonable request.

## Abbreviations

The following abbreviations are used in this manuscript:

AGV	Automatic guided vehicle
AIM	Attribute information model
API	Application programming interface
ASL	Application service layer
BLM	Behavior logic model
CMM	Coordinate measuring machine
CNC	Computer numerical control
CRM	Constraint rule model
CTL	Connection and transmission layer
DT	Digital twin
DTS	Digital twin shop-floor
DT-VSS	Digital twin-based virtual simulation system
FPL	Flexible production line
GPM	Geometric physical model
MES	Manufacturing execution system
NC	Numerical control
New-ICT	New-generation information and communication technologies
PEL	Physical entity layer
PLC	Programmable logic controller
SDK	Software development kit
SQL	Structured query language
TDL	Twin data layer
TM	Twin model
TML	Twin model layer
